# What is the impact of the ACE gene insertion/deletion (I/D) polymorphism on the clinical effectiveness and adverse events of ACE inhibitors? – Protocol of a systematic review

**DOI:** 10.1186/1471-2350-5-23

**Published:** 2004-09-10

**Authors:** M Scharplatz, MA Puhan, J Steurer, LM Bachmann

**Affiliations:** 1 Horten Centre, University of Zurich, Switzerland

## Abstract

**Background:**

The Angiotensin Converting Enzyme (ACE) insertion/deletion (I/D) polymorphism has received much attention in pharmacogenetic research because observed variations in response to ACE inhibitors might be associated with this polymorphism. Pharmacogenetic testing raises the hope to individualise ACE inhibitor therapy in order to optimise its effectiveness and to reduce adverse effects for genetically different subgroups. However, the extent of its effect modification in patients treated with ACE inhibitors remains inconclusive. Therefore our objective is to quantify the effect modification of the insertion/deletion polymorphism of the angiotensin converting enzyme gene on any surrogate and clinically relevant parameters in patients with cardiovascular diseases, diabetes, renal transplantation and/or renal failure.

**Methods:**

Systematic Review. We will perform literature searches in six electronic databases to identify randomised controlled trials comparing the effectiveness and occurrence of adverse events of ACE inhibitor therapy against placebo or any active treatment stratified by the I/D gene polymorphism. In addition, authors of trials, experts in pharmacogenetics and pharmaceutical companies will be contacted for further published or unpublished data. Hand searching will be accomplished by reviewing the reference lists of all included studies. The methodological quality of included papers will be assessed. Data analyses will be performed in clinically and methodologically cogent subgroups. The results of the quantitative assessment will be pooled statistically where appropriate to produce an estimate of the differences in the effect of ACE inhibitors observed between the three ACE genotypes.

**Discussion:**

This protocol describes a strategy to quantify the effect modification of the ACE polymorphism on ACE inhibitors in relevant clinical domains using meta-epidemiological research methods. The results may provide evidence for the usefulness of pharmacogenetic testing for individualised ACE inhibitor therapy.

## Background

Research in genetics and genome sequencing has led to a better understanding of the molecular genetic mechanisms and to the detection of inter-individual genetic differences, so-called polymorphisms, which may have a functional consequence on the response to drugs. Pharmacogenetic tests provide information to better predict and prevent therapeutic failures and adverse drug reaction and raise the hope for an individualised pharmacotherapy [1-3]. Although pharmacogenetic research has moved into several branches of medicine such as cardiology [4], oncology [5] and respiratory medicine [6], the implementation of pharmacogenetic testing into clinical practice is still at the very beginning [7].

The ACE polymorphism identified in 1990 by Rigat and co-workers [8] is one of the best-researched polymorphisms. This polymorphism of the ACE gene is based on the presence or absence of a 287-bp element on intron 16 on chromosome 17. Rigat et al. have shown that the level of circulating ACE enzymes depends on the insertion/deletion (I/D) polymorphism. Since then it has been speculated that these differences in plasma ACE activity associated with the ACE genotype might affect the therapeutic response of ACE inhibitors explaining interindividual variability in cardiovascular or renal response to equivalent doses of ACE inhibitors [9]. Several studies have investigated the extent of this effect modification on response to ACE inhibitors for different indications such as hypertension [10], diabetic nephropathy [11,12] and coronary artery disease [4,13]. There are however inconsistencies in trial findings [14-16] and as a result the extent of effect modification of this polymorphism remains unclear.

Therefore, our objective is to systematically review all randomised controlled trials that assessed to what extent the insertion/deletion polymorphism of the angiotensin converting enzyme gene influences the effect and adverse events of angiotensin converting enzyme inhibitors on any surrogate and clinically relevant parameters in patients with cardiovascular disease, diabetes, renal transplantation and/or renal failure.

## Methods

### Search strategy

We will perform literature searches in (Pre-) MEDLINE (DataStar version, Cary North Carolina), EMBASE (DataStar version, Cary North Carolina), Biosis (Ovid version "Previews 1989 to 2003", New York, New York), the Cochrane Controlled Trials Register (CCTR <3rd Quarter 2003>, Oxford, United Kingdom) and the Science citation index. A preliminary literature search in Medline has been carried out to estimate the range of relevant literature. Out of the citations of the pilot searches (172 citations) we identified articles that met our inclusion criteria. Keywords of these articles were used to refine our search strategies. In collaboration with an information specialist we designed the final search strategies for the six databases avoiding any language restrictions [see additional file 1: the search strategies].

In addition, authors of trials identified in the literature search will be contacted for additional published or unpublished data. Particular efforts will be made to obtain unpublished data on genetic test information and effect measures stratified according to the genetic subtypes examined. We will send our requests and subsequent reminders for additional data to the first and last authors.

Other contacts will include the relevant collaborative review groups of the Cochrane Collaboration, pharmaceutical companies and manufacturers and researchers known to have published pharmacogenetic analyses in the area of cardiovascular disease, diabetes, renal transplantation and/or renal failure. Hand searching will be accomplished by reviewing the bibliographies of all included studies to identify additional relevant articles as well as by using the "related articles" function of PubMed and the citation index of ISI Web of Science.

Anticipating that subgroup analyses investigating gene polymorphisms may not be specifically mentioned in titles or abstracts, we will study the full text of all randomised placebo-controlled trials (RCT) that assessed the effectiveness of ACE inhibitors in order to identify subgroup analyses investigating gene polymorphisms.

### Inclusion criteria

Two reviewers will independently assess all obtained titles and abstracts of the literature search for inclusion. The criteria to be used to identify relevant studies will be 1) randomised controlled trials 2) the investigation of an angiotensin converting enzyme inhibitor used for one of the clinical domains mentioned below and 3) the determination of the deletion/insertion polymorphism of patients.

The two reviewers will then examine the full texts of all potentially relevant citations. The decision on in- and exclusion will be based on the following, more explicit inclusion criteria.

#### Clinical domains

We will include studies investigating ACE inhibitors in the four major clinical domains namely cardiovascular diseases, diabetes, renal transplantation and/or renal failure.

#### Patients

Studies should include patients with the following indications for ACE inhibitor therapy: Heart failure, primary and secondary hypertension, coronary artery disease, diabetic nephropathy, primary nephropathy and status after renal transplantation.

#### Intervention

All licensed or unlicensed ACE inhibitors identified through the literature search will be included.

We will prefer placebo as control intervention in order to study the effect modification. However to assess all available evidence, we will also include pragmatic trials where patients with active treatments (e.g. usual care with any antihypertensive medication) served as controls.

#### Co-intervention

We are mainly interested in studies investigating a single drug exposure with ACE-inhibitors. However we will also include those studies, which allowed co-medications.

#### Description of the pharmacogenetic test

Studies must include a description on how determination of the angiotensin converting enzymes genotypes (DD/ DI/ II) has been performed. If a study does not report details of testing but provides relevant results, authors will be contacted to obtain information of testing technique.

#### Outcomes

We will secure data on any reported outcome, surrogate endpoints (e.g. decrease in blood pressure, changes in hemodynamic parameters, proteinuria, creatinine levels, microalbuminuria) and clinically relevant outcomes (e.g. total and disease specific mortality, morbidity (none fatal myocardial infarction, reinfarction, stroke, transient ischemic attack, rehospitalisation kidney failure or end-stage renal disease)).

The two reviewers will resolve any discrepancies about in- or exclusion by discussion. If agreement cannot be achieved, a third reviewer will make the decision.

### Data extraction strategy

We will use a pre-designed data extraction form that includes different items to assess the studies' external validity [see additional file 2: Data extraction and quality assessment sheet]. Details on study design, treatment, patients and pharmacogenetic tests as well as outcome parameters will be registered onto the data extraction form independently by two reviewers. Also, bibliographic details such as author, journal, year of publication and language, will be registered. This list will be pre-tested on a small sample of included and excluded studies addressing the appraisal topic. A third reviewer will resolve any discrepancies. The data extraction shows the extent of insufficient reporting and authors will be contacted to obtain missing information.

### Quality assessment strategy

All trials included in the review will be assessed using a list of selected quality items indicating components of internal validity and descriptive information [17]. In principle these selected items will enable us to define any process at any stage of inference tending to produce results that differ systematically from the true values (bias) [18]. We will also assess additional methodological aspects that might bias the results of pharmacogenetic studies (e.g. blinding of laboratory assessor of outcomes, blinding of outcome assessor for genotypes, blinding of treatment provider for genotypes. See Data extraction and quality assessment sheet).

In addition, we will assess the description of the methods to determine genotypes. Angiotensin converting enzymes genotypes (DD/ DI/ II) are traditionally determined using polymerase chain reaction (PCR) amplification according to previously published protocols [19]. The D allele is preferentially amplified; therefore each sample found to have the DD genotype should be confirmed in a second independent PCR amplification by the use of an insertion specific primer to avoid the misclassification of the 4–5 percent of samples with DI genotypes as DD genotypes [19]. Beyond the use of the standard PCR with/without the second round of PCR using an insertion-specific primer, there is also a "tri-primer" method, which has been shown to be the proper method to be used in genotyping ACE I/D polymorphism [20]. The methodology of the ACE genotyping will be considered as an explanatory variable for heterogeneity between studies [see additional file 2: Data extraction and quality assessment sheet].

We will pre-test these quality assessment items on a small sample of studies in duplicate and if necessary add missing descriptive items.

Two reviewers will independently score the internal and descriptive validity. The initial degree of discordance between the reviewers will be reported. Discordant scores based on obvious reading errors will be corrected. Discordant scores based on real differences in interpretation will be resolved through consensus. A third party will be sought if necessary. The reviewers will not be blinded for names of authors, institutions, journals or the outcomes of the studies.

These detailed quality assessment will be used to describe the methodological quality of selected studies, to explore potential sources of heterogeneity, to make informed decisions regarding suitability of meta-analysis and to weigh the strength of any conclusions.

### Methods of analysis and synthesis

#### Description of data

The results of the data extraction and assessment of study validity will be presented in different structured tables and in a narrative description [see additional file 2: Data extraction and quality assessment sheet]. This will allow us to display variation in patient characteristics, study quality and results. Thus, the description will include the details about the clinical domain in which the ACE inhibitors have been assessed, information about the study design and quality, a list reporting co-interventions during the study period, details about the study population (baseline characteristics, e.g. severity of disease, ethnic groups, environmental and social characteristics) and a description of the outcome measures that were applied.

Finally the tables will provide the individual study results (all reported outcomes) of the different genotypes in the intervention and control group. Continuous outcomes (e.g. blood pressure) will be summarised in the table as mean differences between baseline and follow up measures. For data of dichotomous outcomes (e.g. cardiovascular death) the relative risks between the results of the DD genotype and the II genotype will be calculated and described in the table. A relative risk of one indicates no difference between two genotypes, where as relative risks lower respectively higher than 1 indicates variations in the treatment effect.

#### Heterogeneity assessment

The heterogeneity assessments help us to examine study characteristics that might be related to variability in the observed outcome. Within each subgroup potential sources of heterogeneity that may affect the imprecision in the estimate of treatment effect such as the study methodology, population characteristics, intensity of intervention, co-medications and risk factors will be examined. We will perform multiple linear regression analyses (meta-regression) to explore sources of between-study heterogeneity. The log transformed odds ratio for dichotomous outcomes (myocardial infarction) and continuous outcomes (blood pressure) measurements will be used as dependent variables and the clinical and methodological items of the extraction sheet as described above will be entered into the model as independent variables.

When a factor is strongly associated with the variation in ln odds ratio or on the continuous outcome, we will stratify the studies on that variable and inspect residual heterogeneity using forest plots.

**Figure 1:**
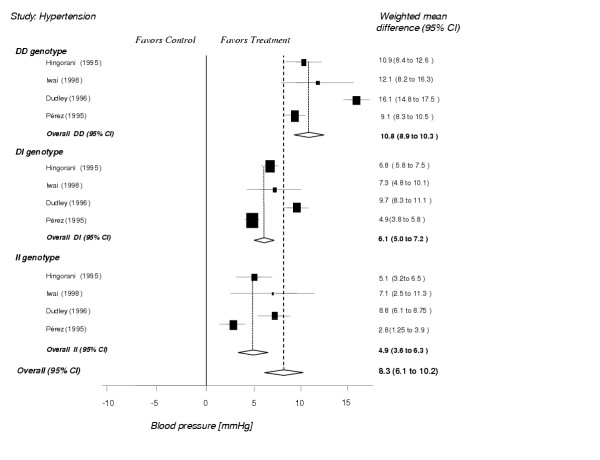
**Example of analysis using virtual data**. *Forest plot: For clinically and methodologically cogent subgroups weighted mean differences (95% confidence interval) for reduction of systolic blood pressure in patients with hypertension have been assessed. This graph displays differences of the ACE inhibitor effect within genotypes. The diamonds below each of the three genotypes indicate the pooled results. The lowest (forth) diamond reflects the overall effect of ACE inhibitors across all genotypes. In this example, the DD genotype shows the largest ACE inhibitor effect and the II genotype shows the smallest effect. The size of the box is related to the number of studied patients.*

If a meta-analysis seems appropriate, that is when the p-value of the chi-squared test for heterogeneity is greater than 0.10, a fixed effects model will be used for pooling. Within clinically and methodologically cogent subgroups relative risks for dichotomous outcomes and weighted mean differences for continuous outcomes will be calculated comparing the contrast between the intervention and the control group within genotypes. The results between the different genotypes will be presented in a forest plot as shown in Figure 1 and differences will be statistically assessed.

The pooled results of a cogent subgroup will produce an estimate of the differences in the average effect of ACE inhibitors observed between ACE genotypes. Thus the treatment effect of each genotype could be compared to the overall effect of ACE inhibitors regardless of the genotype.

All statistical analyses will be performed using the Stata statistical software package (StataCorp. 2004. Stata^® ^Statistical Software: Release 8.2 College Station, Texas, USA).

## Discussion

This review shows an efficient approach to quantify the effect modification of the ACE polymorphism on ACE inhibitors when applied in different clinical occasions. We aim to resolve part of the controversy in the literature by quantifying the influence of the three genotypes (DD/DI/II) on different outcomes and in the light of study methodology and participants characteristics. These results should inform clinicians about the potential of pharmacogenetic testing to individualise ACE inhibitor treatment.

## Competing interests

None declared.

## Authors' contributions

LMB, JS and MS initiated the project. MS wrote the first draft of the protocol. MP, JS and LMB critically reviewed and revised the manuscript. All authors approved the manuscript.

## Pre-publication history

The pre-publication history for this paper can be accessed here:



## Supplementary Material

Additional File 1 Search strategies: Search terms and number of citations listed for five electronic databases Click here for file

Additional File 2 Data extraction and quality assessment sheet: Example of data extraction and quality assessment for the study of Hernandez [21] Click here for file
