# Supplementary material for: What is the impact of the ACE gene insertion/deletion (I/D) polymorphism on the clinical effectiveness and adverse events of ACE inhibitors? – Protocol of a systematic review
Source: BMC Med Genet. 2004 Sep 10;5:23. doi: 10.1186/1471-2350-5-23 (PMC518966; doi:10.1186/1471-2350-5-23)
Supplement: Additional File 1 — Search strategies: Search terms and number of citations listed for five electronic databases [file 1471-2350-5-23-S1.doc]

## Appendix 1: Search strategies

The pilot search in MEDLINE identified relevant articles using the following keywords Angiotensin converting enzyme inhibitor, peptidyl-dipeptidase A, genetics, pharmacogenetic, polymorphism, deletion, insertion, genotype and phenotype.

#### **MEDLINE and EMBASE (Data Star® interface)**

###### **Search strategy**

**Medline Terms Number of citations Search term**

1 6584 PEPTIDYL-DIPEPTIDASE-A#
2 1816 1 WITH GE
3 1272 1 AND POLYMORPHISM$
4 26395 ANGIOTENSIN ADJ CONVERTING ADJ ENZYME
5 1071 4 AND POLYMORPHISM$
6 2064 2 3 5
7 26542 ANGIOTENSIN-CONVERTING-ENZYME-INHIBITORS#
8 16349 ENALAPRIL$ CAPTOPRIL$ RAMIPRIL$
9 1281 QUINAPRIL$ FOSINOPRIL$ BENAZEPRIL$
10 958 CILAZAPRIL$ TRANDOLAPRIL$
11 2535 PERINDOPRIL$ LISINOPRIL$
12 20422 ANGIOTENSIN ADJ CONVERTING ADJ ENZYME ADJ INHIBIT$5
13 31169 7 8 9 10 11 12
14 265 6 AND 13

**Embase Terms Number of citations Search term**

15 9008 DIPEPTIDYL-CARBOXYPEPTIDASE#
16 1282 15 AND GENETIC-POLYMORPHISM#
17 17425 ANGIOTENSIN ADJ CONVERTING ADJ ENZYME
18 959 17 AND POLYMORPHISM$
19 1464 16 18
20 53524 DIPEPTIDYL-CARBOXYPEPTIDASE-INHIBITOR#
21 30794 ENALAPRIL$ CAPTOPRIL$ RAMIPRIL$
22 3375 QUINAPRIL$ FOSINOPRIL$ BENAZEPRIL$
23 2275 CILAZAPRIL$ TRANDOLAPRIL$
24 6441 PERINDOPRIL$ LISINOPRIL$
25 8875 ANGIOTENSIN ADJ CONVERTING ADJ ENZYME ADJ INHIBIT$5
26 54776 20 21 22 23 24 25
27 266 19 AND 26

28 531 COMBINE 14,27
29 94 DUPLICATES FROM STATEMENT 28
30 437 KEPT FROM STATEMENT 28

#### **BIOSIS Previews <1989 to 2003 Week 45> (OVID® interface)**

###### **Search strategy and number of citations in brackets**

1 angiotensin converting enzyme.af. (23214)
2 9015-82-1.rn. (12258)
3 1 or 2 (23836)
4 polymorphism$.af. (83910)
5 3 and 4 (1866)
6 (enalapril$ or captopril$ or ramipril$).af. (13109)
7 (quinapril$ or fosinopril$ or benazepril$).af. (1734)
8 (cilazapril$ or trandolapril$).af. (1172)
9 (perindopril$ or lisinopril$).af. (2685)
10 exp angiotensin-converting enzyme inhibitor-drug/ (5276)
11 angiotensin converting enzyme inhibit$.af. (15017)
12 or/6-11 (24342)
13 5 and 12 (187)

#### **COCHRANE CENTRAL REGISTER OF CONTROLLED TRIALS (OVID® interface)**

###### **Search strategy and number of citations in brackets**

1 (angiotensin$ and converting and enzyme).af. (2504)
2 polymorphism$.af. (493)
3 1 and 2 (49)
4 enalapril$.af. (1599)
5 captopril$.af. (1649)
6 ramipril$.af. (302)
7 quinapril$.af. (196)
8 fosinopril$.af. (107)
9 benazepril$.af. (124)
10 cilazapril$.af. (203)
11 trandolapril$.af. (134)
12 perindopril$.af. (261)
13 lisinopril$.af. (510)
14 angiotensin converting enzyme inhibit$.af. (1568)
15 or/4-14 (4996)
16 3 and 15 (17)

#### **SCIENCE CITATION INDEX (ISI web of Science)**

Web of Science will be searched using the following terms:

Topic=angiotensin same converting same enzyme and polymorphism* and (enalapril* or captopril* or ramipril* or quinapril* or fosinopril*or benazepril* or cilazapril* or trandolapril* or perindopril* or lisinopril* or angiotensin same converting same enzyme same inhibit*); DocType=All document types; Language=All languages; Databases=SCI-EXPANDED; Timespan=All Years; (sorted by latest date)
